# Neurodevelopmental Disorder with Psychomotor Delay, Hearing Loss, and Spasticity Caused by Compound Heterozygous *SPATA5L1* Variants—Expanding Phenotype

**DOI:** 10.3390/jcm14238442

**Published:** 2025-11-28

**Authors:** Artur Polczyk, Ewelina Wolańska, Anna Zimny, Agnieszka Zubkiewicz-Kucharska, Mateusz Biela, Agnieszka Pawelak, Robert Śmigiel

**Affiliations:** 1 Medical Education and Simulation Laboratory, University Centre of Physiotherapy and Rehabilitation, Faculty of Physiotherapy, Wroclaw Medical University, 50-368 Wroclaw, Poland; 2 Department of Clinical Physiotherapy and Rehabilitation, University Centre of Physiotherapy and Rehabilitation, Faculty of Physiotherapy, Wroclaw Medical University, 50-368 Wroclaw, Poland; 3 Department of General and Interventional Radiology and Neuroradiology, Wroclaw Medical University, 50-368 Wroclaw, Poland; 4 Department of Pediatrics, Endocrinology, Diabetology and Metabolic Diseases, Faculty of Medicine, Wroclaw Medical University, 50-367 Wroclaw, Poland; agnieszka.zubkiewicz-kucharska@umw.edu.pl (A.Z.-K.); mateuszbiela14@gmail.com (M.B.); robert.smigiel@umw.edu.pl (R.Ś.); 5 Department of Genetics, Wroclaw Medical University, 50-368 Wroclaw, Poland

**Keywords:** *SPATA5L1* gene, neurodevelopmental disorders, psychomotor disorders, hearing loss, sensorineural, cerebral palsy, hypotonia, whole exome sequencing, vojta method, physiotherapy

## Abstract

**Background:** *SPATA5L1*-related neurodevelopmental disorder is a recently described condition characterized by psychomotor delay, sensorineural hearing loss, and variable motor dysfunction. Because only a few cases have been reported, the full phenotypic spectrum remains poorly defined. Expanding clinical characterization is crucial for improving early diagnosis and targeted management. **Case Presentation:** We report a 24-month-old female with compound heterozygous *SPATA5L1* variants c.1918C>T (p.Arg640Ter) and c.2066G>T (p.Gly689Val). She presented with global psychomotor delay, bilateral sensorineural hearing loss, strabismus, and craniofacial dysmorphism. Brain MRI showed cortical and white matter atrophy, delayed myelination, and a thin corpus callosum. Vojta neurodevelopmental assessment demonstrated an 11-month motor delay, abnormal responses in all seven Vojta postural reactions, and persistent primitive reflexes. Early EEG recordings were without significant changes, whereas abnormalities emerged later in the clinical course. Genetic testing confirmed the variants in trans. **Management and Outcomes:** Early rehabilitation including reflex locomotion therapy was initiated. The persistence of primitive reflexes, central hypotonia, and pathological postural reactions provided a coherent neuromotor profile and indicated a high vulnerability to atypical motor development, and do not rule out the possibility of later evolution toward a spastic–dystonic motor pattern. These findings, combined with neuroimaging abnormalities, refined the patient’s neuromotor phenotype and guided individualized therapeutic planning. **Conclusions:** This case expands the clinical and neurodevelopmental spectrum associated with *SPATA5L1* variants and highlights the diagnostic value of integrating genomic sequencing with structured motor assessments. Early, multidimensional evaluation may improve recognition of rare neurodevelopmental disorders and support more precise prognostication and rehabilitation strategies.

## 1. Introduction

The spermatogenesis-associated 5-like 1 (*SPATA5L1*, currently known as *AFG2B*, OMIM: 619578) gene is classified as an orphan gene, encoding a protein whose precise function remains incompletely understood [1]. It belongs to the AAA+ ATPase family, a group of proteins essential for cellular homeostasis and genome stability, including mitosis, DNA replication and repair, metabolism, and replisome proteostasis [2]. *SPATA5L1* forms a heterodimeric AAA+ ATPase complex with SPATA5 and its partners C1orf109-CINP (55LCC complex), which interacts with replisome-associated factors and participates in maintaining proteostasis during DNA replication. Structural studies have shown that the N-terminal domains of *SPATA5* and *SPATA5L1* bind C1orf109-CINP, forming a funnel-like structure over the ATPase motor [2].

Expression analyses demonstrate that *SPATA5L1* is enriched in neurons and glial nuclei, with notable expression also reported in neurosensory hair cells of the inner ear [1]. Transcriptomic data further suggest its involvement in cell adhesion receptor signaling and focal adhesion processes [1]. Importantly, biallelic pathogenic variants in *SPATA5L1* were only recently recognized as a cause of a distinct neurodevelopmental disorder, and fewer than 30 affected individuals have been described to date [1,3,4]. Reported core clinical manifestations include global developmental delay, intellectual disability, spastic–dystonic cerebral palsy, epilepsy, and nearly universal sensorineural hearing loss [1]. Craniofacial dysmorphism, microcephaly, and cortical visual impairment appear in a subset of cases, while brain MRI frequently reveals global cerebral volume loss, delayed myelination, and thinning of the corpus callosum [1,3,4].

*SPATA5* and *SPATA5L1* are paralogous genes with high sequence similarity and shared biochemical function, yet their expression profiles differ, and clinical phenotypes show overlapping but distinct patterns [2,5]. SPATA5-related disorder (NEDCAS) typically features earlier seizure onset, more severe microcephaly, and more frequent movement disorders [5], whereas SPATA5L1-associated disease more consistently presents with spastic–dystonic motor features and sensorineural hearing loss [1]. Despite these insights, the genotype–phenotype correlations remain poorly defined, largely due to the rarity of reported cases and the diversity of variant types.

Given these gaps, each well-characterized case contributes meaningfully to the understanding of SPATA5L1-associated disease. Detailed functional motor phenotyping—particularly using structured neurodevelopmental evaluation tools such as the Vojta method—has not been systematically reported in previous cases and may help refine the clinical boundaries of this disorder.

Therefore, the aim of this case report is to expand the clinical and neurodevelopmental phenotype associated with biallelic *SPATA5L1* variants by integrating genomic findings with detailed functional motor assessment, neuroimaging, and early developmental evaluation.

## 2. Case Report

### 2.1. General Medical History

The female patient was born from a first, uneventful pregnancy of non-consanguineous parents, delivered by cesarean section due to maternal psychiatric indications. The prenatal course was unremarkable, with normal fetal movements and ultrasound findings. At birth, the newborn presented with normal anthropometric parameters (weight 3330 g, length 50 cm, head circumference 35 cm) and Apgar scores of 10/10. Neonatal adaptation was normal, with adequate feeding coordination; initial breastfeeding was partially supplemented with bottle feeding, and exclusive formula feeding was introduced at approximately 7 months of age.

At four months of age, the parents expressed concerns regarding their daughter’s psychomotor development, noting an inability to support herself in the prone position, limited eye contact, and the absence of voluntary grasping. As a result, early rehabilitation was initiated in a specialized center. Due to unsatisfactory progress during the rehabilitation process and the presence of dysmorphic features, the physiotherapist recommended further extended diagnostics. In the 7th month of life audiological evaluation revealed bilateral sensorineural hearing loss of 60 dBnHL, and hearing aids were fitted. At approximately 9 months of age, cranial ultrasound was normal, while neurological examination revealed decreased central muscle tone, delayed psychomotor development, and positional asymmetry with asymmetric cutaneous reflexes, including asymmetrical superficial reflex responses such as the plantar and palmar grasp reflexes, which were more active on the left side. At 9 months, physical examination revealed a eutrophic infant (10 kg, OFC 43 cm) with craniofacial dysmorphism, including bitemporal narrowing, wide mouth, and epicanthal folds. In this time EEG performed for suspected seizures showed no epileptiform activity. At 11 months and two weeks of age, in the quantitative and qualitative assessment consistent with the Vojta diagnostic method, the patient showed no improvement in spontaneous motor activity compared with the previous evaluation. In the reflex assessment, a persistently strong Moro response was observed, along with continued activity of the orofacial rooting and the Babkin reflex. All seven Vojta postural reactions were evaluated as abnormal. Deep tendon reflexes were assessed by the neurologist as symmetric, and no pathological plantar responses from the Babinski group were present. At the age of 12 months ophthalmologic and optometric assessment demonstrated intermittent divergent strabismus of the right eye, hyperactivity of the inferior oblique muscle, sluggish pursuit, and hypermetropia with astigmatism bilaterally; anterior segment and fundus examinations were unremarkable.

### 2.2. Assessment of the Patient’s Psychomotor Development

**Figure 1:**
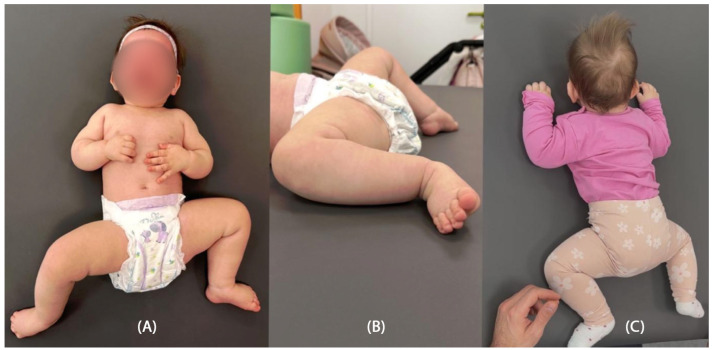
Delayed spontaneous motor skills of the patient in the supine position (**A**), with a constant tendency for lateral flexion and rotation to the right, also in the prone position (**C**), with excessive flexion and abduction in the hip joints and pronation of the ankle joints (**B**).

Psychomotor development assessment according to the Vojta neurodevelopmental diagnostics method was conducted at a calendar age of 14 months. In the initial phase of the study, the patient was wearing hearing aids. She established short-term pattern contact with the diagnostician, and occasionally babbled. The patient is unable to turn independently from a supinated position to lying on her front, does not show any desire to grab. Quantitatively, the girl presents patterns from around the completed 12th week of life, trying to build a stable position of lying on her back (Figure 1A). Qualitative deviations that make it difficult for the patient to resist gravity in lying on her back and front are associated with reduced muscle tone in the center, which is manifested by hyperabduction of constantly passively flexed hip joints, flexion of the knee joints, and a tendency to pronate the feet in the lower ankle joints (Figure 1B). The patient tries to bring both hands to the midline, but does not yet do it under visual control. She is able to briefly follow the stimulus by rotating her head to a limited extent to both sides, with a greater range of rotation to the right, which also affects the more intensive loading of the right side, with its elongation in the supine position. In the frontal position, the patient is unable to independently obtain support functions, straightens the shoulder joints, flexing and relieving the elbows. Assisted and after reflex locomotion therapy, she briefly maintains support independently. In the frontal position, the rotation of the cervical and thoracic spine is also expressed to the right, which affects the asymmetric distribution of body weight, with more intensive loading of the left side of the body (Figure 1C).

**Figure 2:**
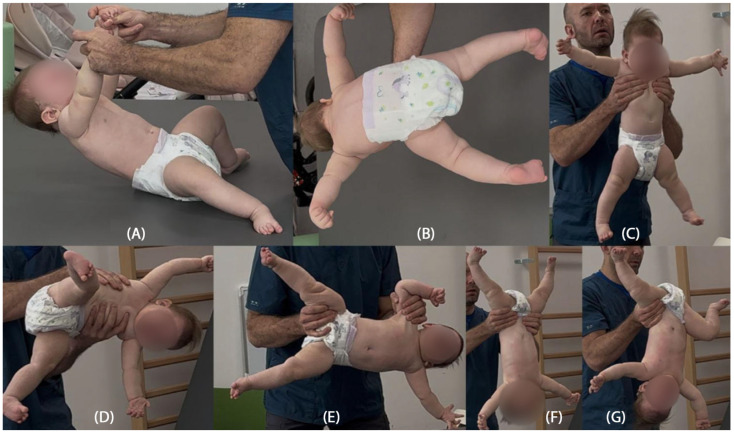
Seven body positioning reactions in space: (**A**) traction test with visible partial reaction in the head and cervical spine towards flexion, with excessively expressed abduction and flexion in the hip joints and flexion in the knee joints with pronation of the ankle joints; (**B**) Landau reaction, (**C**) axillary reaction, (**D**) Vojta reaction, (**E**) Collis horizontal reaction, (**F**) Peiper-Isbert reaction, (**G**) Collis vertical reaction. In all reactions except the traction test, the Moro reflex was clearly preserved, with inadequate response in the upper limbs towards abduction with blocking of the elbow joint in extension, abduction position of the hip joint and lack of support reactions in the lower lying limbs in the horizontal Collis reaction. Due to both the quality of partial responses and the quantitative delay, each of the seven responses was scored as incorrect.

In seven postural reactions of the body in space according to Vojta, the patient presents a persistent Moro reflex in six of the seven postural tests, with dominant hyperabduction in the hip joints and an extension tendency of the cervicothoracic segment of the spine, together with right-sided rotation (Figure 2B–G). Only in the first suspension (traction) test does the patient show a partial, age-appropriate flexion response of the head and cervicothoracic spine, opposing gravity by activating the ventral neck and thoracic muscles; in the lower limbs, excessive abduction, hip and knee flexion, and foot pronation remain constantly visible (Figure 2A).

The structured neurodevelopmental assessment based on the Vojta diagnostic method was a key element in the clinical interpretation of this case. This tool enables both qualitative and quantitative evaluation of spontaneous motor activity and postural reactions, allowing early identification of abnormal motor patterns. A particularly significant finding was the persistently present Moro reaction across six of the seven postural tests, accompanied by rigid extension patterns of the limbs. These abnormalities were evident from the beginning of the rehabilitation process and consistently raised concern for atypical motor organization. The postural reactions illustrated in the provided figures had been assessed earlier in therapy and the same pathological features remained clearly visible, demonstrating the persistence and stability of the abnormal motor patterns over time. The lack of clinical progress despite therapy, together with the persistent, clearly inappropriate responses in the seven postural reactions, prompted the physiotherapist to recommend further diagnostic evaluation, including comprehensive genetic testing. Furthermore, the Vojta method offers an objective and reproducible framework for monitoring therapeutic progress, allowing consistent assessment of changes in motor function over time.

### 2.3. Genetic Evaluation

The patient was referred for a genetic evaluation of the dysmorphic syndrome and psychomotor and social neurodevelopment delay, as well as bilateral hearing loss. Whole Exome Sequencing (TRIO_WES) was performed, extended with the analysis of the full mitochondrial genome and a panel of known pathogenic variants described in the ClinVar database located outside the coding sequence.

DNA copy number variation (CNV) analysis did not reveal any changes that would explain the symptoms presented by the patient. The presence of uniparental disomy was also not demonstrated.

Bioinformatic analysis covering variants with a known or potential impact on protein/RNA coding revealed the presence of variants c.1918C>T and c.2066G>T of the *SPATA5L1* gene in a heterozygous status. Analysis of the segregation of the indicated variants in the *SPATA5L1* gene indicates their inheritance from parents (in trans scheme)—in the proband we observe a configuration of a compound heterozygote, and in the parents the carrier state: variant c.1918C>T in the father and c.2066G>T in the mother, respectively. Both variants of the *SPATA5L1* gene are described in the literature in connection with intellectual disability, cerebral palsy of a spastic-dystonic nature, epilepsy and hearing loss [1,3,4]. The c.1918C>T variant of the *SPATA5L1* gene is destructive in nature, resulting in premature occurrence of the “stop” codon. In the ACMG classification it is assessed as having uncertain significance. The c.2066G>T variant of the *SPATA5L1* gene is of the type of missense change, according to the ACMG classification it is assessed as probably pathogenic. The *SPATA5L1* gene variant c.2066G>T has been reported in the ClinVar database as having pathogenic significance. The c.1918C>T nonsense variant fulfills the PVS1 criterion as a null alteration in a gene in which loss-of-function is an established pathogenic mechanism, while its rarity (PM2), confirmed trans configuration with a second pathogenic/likely pathogenic allele (PM3), and the proband’s phenotype consistent with SPATA5L1-related disease (PP4) support its clinical relevance. The missense variant c.2066G>T (p.Gly689Val), which results in a non-conservative amino acid substitution within the SPATA5L1 protein, is likewise very rare (PM2) and occurs in *trans* with a loss-of-function allele in a recessive disorder (PM3), with a clinical phenotype matching previously reported cases (PP4) [6]. Pathogenic *SPATA5L1* gene variants have been associated with neurodevelopmental disorders with hearing loss and spasticity with an autosomal recessive pattern of inheritance (OMIM: 619616) [1].

Additionally, analysis for all de novo or mosaic variants in the parent also revealed the c.314G>A variant in the *FRYL* gene in a heterozygous de novo pattern in the proband. The identified *FRYL* gene variant may affect the transcript splicing process and is described in the literature as having a possible impact on the development of neurodevelopmental disorders in patients. Due to the origin of wide-field genome sequencing data with low coverage and the disproportion of the obtained reads from both sequencing directions, it cannot be ruled out that this is an artifact, and the ambiguous association of *FRYL* gene damage with human diseases is difficult to prove [7].

### 2.4. Brain Imaging and Follow-Up

**Figure 3:**
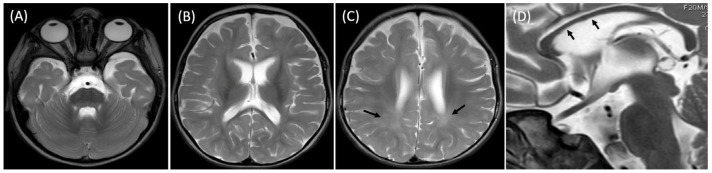
Axial T2-weighted brain MR images (**A**–**C**) showing anterior temporal hypoplasia (**A**) and widened pericerebral fluid spaces (**B**,**C**) indicating cortical volume loss as well as mild ventriculomegaly (**B**,**C**) consistent with white matter atrophy and periventricular T2-hyperintensities (**C**, arrows) suggestive of delayed white matter myelination. Sagittal T2 weighted image (**D**) showing thin corpus callosum (arrows).

At 20 months, the brain MRI revealed mild generalized atrophy of the cortex and the white matter, bilateral T2 hyperintensities of the posterior portions of the white matter indicating its delayed myelination and slightly hypoplastic corpus callosum (Figure 3).

Before the child reached two years of age, the parents observed brief episodes lasting several to several dozen seconds, characterized by a moment of unresponsiveness, tightening of the hands, followed by loud laughter. According to the mother, the symptoms intensified after increasing the volume of the hearing aids and also tended to occur when the child was tired or sleep-deprived. These episodes could appear daily or several times per day. Recently, a new type of event emerged—sudden body jerks accompanied by upward and sideways deviation of the eyes. The EEG performed at approximately 2 years and 2 weeks of age showed abnormalities during wakefulness and sleep, including bilateral sharp waves, incomplete FO–FW complexes, and theta activity. Although the later EEG demonstrated epileptiform abnormalities, no clinically confirmed epileptic seizures have been documented to date. On physical examination head circumference measured 44.5 cm (below the 3rd percentile). The child was engaged and interested in her surroundings. Marked hypotonia was still present; however, pronounced upper-limb stiffening was observed, with a ‘slipping’ or ‘clunking’ phenomenon in the shoulder joints, more pronounced on the left side. A summary of the clinical observations to date, including spontaneous motor skills, reflexes and the assessment of seven postural responses, is presented in Table 1.

## 3. Discussion

The clinical presentation of our patient with *SPATA5L1* variants shows considerable overlap with previously reported cases of *SPATA5L1*-related neurodevelopmental disorders characterized by hearing impairment and neurological manifestations [1,3,4].

Biallelic variants in the *SPATA5L1* gene were only recently recognized as a cause of a distinct neurodevelopmental phenotype. Previous reports emphasize that most pathogenic variants in *SPATA5L1* are private and occur rarely [1]. However, an identical variant, c.2066G>T, has been reported in four individuals within a cohort of 25 patients described by Richard et al. [1], all of whom exhibited profound developmental delay and sensorineural hearing loss. The same variant was also detected in our patient, highlighting its recurrence and potential clinical significance. In combination with the rarely reported nonsense variant c.1918C>T, the compound heterozygous state may account for the observed clinical phenotype. Our findings, consistent with previous observations, expand further the spectrum of known variant configurations in this gene. Nevertheless, the currently available cohort described in the literature remains too small to allow reliable associations between specific variants and clinical manifestations or to support genotype–phenotype correlations.

The clinical picture typically combines psychomotor delay, sensorineural hearing loss, spastic-dystonic cerebral palsy, epilepsy, and variable craniofacial dysmorphism [1]. Our patient demonstrates most of these cardinal features, including bilateral hearing loss, global psychomotor delay, abnormal neurodevelopmental reflexes, and brain MRI abnormalities. Interestingly, despite the presence of two pathogenic variants, epilepsy was not observed during the reported period, highlighting the phenotypic variability of *SPATA5L1*-associated disease.

In contrast to the published cohort, in which most individuals with bi-allelic *SPATA5L1* variants exhibited spasticity and/or dystonia as predominant motor features [1], our patient at 14 months of age demonstrated generalized hypotonia with markedly delayed psychomotor development, without clinical signs of hypertonia to date. Similarly to previously reported cases, she presented with bilateral moderate-to-severe sensorineural hearing loss, diagnosed in infancy. Epileptic seizures, which are described in a substantial proportion of affected individuals [1,3] have not been confirmed in our patient, as EEG recordings performed for suspected episodes revealed no epileptiform activity. Craniofacial dysmorphic features were noted, including bitemporal narrowing, wide mouth, and epicanthal folds, partially overlapping with those reported in approximately one-third of affected individuals in the literature. Ophthalmologic examination additionally revealed intermittent strabismus and refractive errors, which are not systematically described in prior cohorts.

Overall, while the clinical picture of our patient is consistent with the core phenotype of developmental delay, hypotonia, hearing impairment, and craniofacial dysmorphism, the absence of seizures and spastic-dystonic features at this stage may reflect either her young age or an expansion of the phenotypic spectrum associated with *SPATA5L1*-related disease.

Neuroimaging findings in our patient, including cortical atrophy, delayed myelination, and a hypoplastic corpus callosum, are consistent with previously reported MRI patterns [1]. These structural abnormalities reflect the central role of *SPATA5L1* in neuronal maintenance. Biochemical studies indicate that SPATA5L1 protein, together with SPATA5, forms the heterohexameric AAA+ ATPase complex (55LCC), which safeguards replisome proteostasis and ensures genome integrity [2].

Recent reviews of neurogenetic disorders with hearing loss also highlight AFG2A and AFG2B as paradigmatic examples of genes linking DNA replication stress and combined neurodevelopmental–auditory phenotypes [8].

Missense variants such as p.Gly689Val are predicted to disrupt ATP binding and hydrolysis, thereby impairing the catalytic function of the ATPase motor [2]. This molecular mechanism provides a plausible link between genotype and neurodevelopmental pathology.

Neurogenetic disorders with combined developmental and auditory phenotypes, including *AFG2A (SPATA5)* and *AFG2B (SPATA5L1*)–related syndromes, are increasingly recognized within a broader spectrum of neurogenetic hearing loss [8].

Comparison with the paralogous gene *SPATA5* highlights both shared and divergent disease mechanisms. *SPATA5*-related disorder (NEDCAS) presents with earlier seizure onset, more pronounced microcephaly, and frequent movement disorders [5], whereas *SPATA5L1*-associated cases appear to manifest more consistently with spastic-dystonic cerebral palsy and hearing loss [1]. This divergence supports the view that, although functionally interconnected within the same ATPase complex, *SPATA5* and *SPATA5L1* proteins exert partially distinct roles in neuronal biology.

The incidental detection of a de novo *FRYL* variant in our patient introduces further complexity. *FRYL* has been associated with neurodevelopmental delay and dysmorphic features [7], but its role remains incompletely defined, and technical sequencing artifacts cannot be excluded. At present, *FRYL* gene cannot be considered a major contributor to this patient’s phenotype, though potential modifying effects cannot be ruled out.

Published data, particularly the cohort described by Pan et al., indicate that pathogenic *FRYL* variants are usually associated with more severe phenotypes, including developmental delay/intellectual disability, central nervous system anomalies, psychiatric conditions, dysmorphic features, shortened long bones (mesomelia), as well as cardiovascular, genitourinary, gastrointestinal, and ocular anomalies [7]. Similar features were also reported in the case study by Singh et al., further supporting the view that *FRYL*-related disorders are typically associated with complex and multisystem involvement [3,9]. In contrast, our patient presents with a comparatively milder clinical picture. It remains possible that splice-affecting variants, such as the one identified here, may lead to attenuated manifestations relative to truncating or missense variants, although current evidence is limited to only a few published reports. As additional cases are documented, clearer genotype–phenotype correlations may emerge, particularly regarding differences between variant types; at present, however, such associations remain hypothetical.

This case illustrates the diagnostic value of combining advanced genomic methods with detailed functional neurodevelopmental assessments. The Vojta method enabled a precise quantification of motor delay and revealed a persistent Moro reaction, orofacial rooting and Babkin reflex, as well as asymmetric cutaneous reflexes, thereby supporting the clinical diagnosis while also providing a standardized tool for assessing psychomotor development and evaluating therapeutic effects or the lack thereof. Notably, recent data by Podstawski et al. (2025) in Children (Vol. 12, 976) demonstrate that persistent primitive reflexes, postural asymmetry and altered postural reactions as assessed via the Vojta method significantly correlate with central coordination disorders in infants at risk of atypical development—which reinforces our observation that integrating structured Vojta assessment with genomic diagnostics may allow earlier and more accurate detection of motor phenotype deviations [10]. In line with this, Futagi et al. (2013) reported that persistence of the Babkin reflex beyond the expected age of integration may reflect delayed maturation of brainstem–cortical pathways and may signal an increased risk for underlying neurodevelopmental disorders, further underscoring the diagnostic weight of our findings [11]. Such integrative approaches improve the recognition of rare genetic syndromes, guide early rehabilitation strategies, and inform genetic counseling. In conclusion, our case broadens the phenotypic spectrum of *SPATA5L1*-related disease, confirms its overlap with but distinction from *SPATA5*-related disorder, and emphasizes the need for early, multidimensional assessment in children presenting with unexplained psychomotor delay.

## Figures and Tables

**Table 1:** Summary of spontaneous motor activity, primitive reflexes, and postural reactions.

Domain	Findings	Interpretation
Spontaneous motor activity	Persistent central hypotonia with hip hyperabduction; upper-limb stiffening with a ‘clunking’ shoulder phenomenon (left > right); spontaneous motor patterns at ~12 weeks; difficulty counteracting gravity in supine and prone; absent rolling; seizure-like episodes reported before age of two	Marked delay in spontaneous motor development with persistent qualitative abnormalities and sustained features of central hypotonia.
Primitive reflexes	Persistent strong Moro reflex; persistent orofacial rooting reflex; persistent Babkin reflex; symmetric deep tendon reflexes; no pathological plantar responses (Babinski group).	Abnormal persistence of primitive reflexes at 11 months of age, indicating delayed maturation of supraspinal control; absence of Babinski sign suggests no pyramidal release.
Seven Vojta postural reactions	All 7 Vojta postural reactions were abnormal at both the 11-month and 14-month assessments.	Severe central coordination disorder: 7/7 pathological postural reactions, indicating a significant deviation from the typical motor phenotype

## Data Availability

The original contributions presented in this study are included in the article. Further inquiries can be directed to the corresponding authors.
